# Oculohypotensive effects of various acetozolamide nanopreparations for topical treatment of animal model-induced glaucoma and their impact on optic nerve

**DOI:** 10.1371/journal.pone.0212588

**Published:** 2019-02-21

**Authors:** Sherif S. Mahmoud, Eman S. ElAbrak, Mervat A. Aly, Eman M. Ali

**Affiliations:** Biophysics and Laser Science Unit, Research Institute of Ophthalmology, Giza, Egypt; University of Illinois at Chicago, UNITED STATES

## Abstract

Acetozolamide-ACZ, carbonic anhydrase inhibitor- is still the most effective systemic drug for glaucoma treatment. Due to its limited ocular bioavailability, topical formulations are not available yet. This study introduces within the framework of nanotechnology three nanopreparations of acetozolamide for topical application, one of them is liposomal phospholipid vehicle and the other two preparations are propolis and *Punica granatum* (pomegranate). The hypotensive effect of these different nanopreparations in lowering the increased intraocular pressure that was induced in experimental rabbits is monitored for 130 hrs. Structural characteristics of the optic nerve dissected from all involved groups were studied by Fourier transfrom infrared spectroscopy. The obtained results indicate the impact of the topically applied acetozolamide nanopreparations in lowering the intraocular pressure to its normotensive control value. On the other hand, the optic nerve characteristics were found to be dependent on the way acetozolamide introduced. Glaucoma affects structural components that contain OH group and increases β-turns of the protein secondary structure while, reducing the content of both α-helix and Turns. In the same context, liposomal-acetozolamide and propolis nanopreparations protecting the optic nerve protein secondary structure from these changes associated with glaucoma.

## Introduction

Glaucoma by definition is a group of diseases that damage the eye's optic nerve leading to loss of vision and results in blindness. The standard and usual treatment of glaucoma includes eye drops, laser trabeculoplasty, or conventional surgery where, the treatment strategy is based on lowering and/or controlling the intraocular pressure. Aging is among the several reasons for developing glaucoma and the prevalence of glaucoma rises as the population got older; furthermore, increased oxidative stress is associated with aging[1]. Since glaucoma is the second leading cause of blindness worldwide[2], there is an insist need for developing a new topical-treatment modality that can lower the IOP and on the other hand protect the optic nerve from being damaged.

Acetozolamide is carbonic anhydrase inhibitor drug that physiologically acts through inhibiting water permeability of membranes by interacting with aquaporins, and its ability of lowering the IOP is attributed to reduce the production of aqueous humor in the ciliary body. Its systemic administration is associated with side effects such as central nervous system depression, renal failure, diuresis, vomiting, anorexia, and metabolic acidosis[3–4].

Epidemiological evidences support the correlation between plant phytochemicals and the beneficial health effects as reduced risk of cardiovascular disease [5], anticancer activity[6]and antimicrobial/anti-inflamatory properties[7]. The interest of using complementary and alternative medicine by glaucoma patient is increased[8]and plant phytochemicals were found to have antiglaucoma properties[9–10].

Since 1981, the contribution of reactive oxygen species in damaging both the trabecular meshwork (cells that responsible for draining the aqueous humor from the anterior chamber) and the head of the optic nerve was postulated and confirmed [11–12]. Plant extracts are rich in bioactive compounds with antioxidant properties as polyphenols and flavonoids.

Oral administration of ACZ in rabbits (5 mg/kg body weight) twice a day for two weeks significantly reduces the expected rise of the IOP [13]; in addition, liposomes as a carrier for antiglaucoma drugs were previously studied [14–17].Therefore, the aim of this study, in one hand, is to apply the concepts of nanoscience in terms of liposomes and polyphenols rich-nanopreparations to produce topical-ocular acetozolamide preparations, and on the other hand to evaluate their possible neuroprotection effect(s) on the optic nerve by monitoring its structural characteristics by Fourier transfrom infrared spectroscopy (FTIR).

## Materials and methods

### Materials

Chinchilla rabbits of both sex and weighing 2.5–3 Kg were selected from the animal house facility at the research Institute of Ophthalmology (Giza, Egypt). Rabbits were housed individually in cages at 27°C, relative humidity 60–70%, and a 12 hrs light cycle; they have access to water and food ad- libitum. Propolis powder was obtained from the ministry of agriculture production facility at Shoubra El Khema district (Cairo, Egypt) during Mar 2018 while, *Punica granatum* (pomegranate) peel powder was obtained from local herbalist store (El-Hamzawey store for medical herbes) in Cairo, Egypt. Acetozolamide (ACZ) powder ((N-(5-Sulfamoyl-1,3,4-thiadiazol-2-yl)acetamide), ≥99% purity),L,α-dipalmitoylphosphatidylcholine (DPPC), the hexane-organic solvent (99%) and potassium bromide (KBr-IR grade for infrared spectroscopy) were purchased from Sigma-Aldrich (St. Louis, MO, USA). Betamethasone suspension (Diprofos) was obtained in the form of ampoules from SEDICO drug Industries Company, October city, Egypt. The muscle relaxant drug, Ketamine hydrochloride (Ketalar), was purchased from Pfizer Inc. (New York, NY, USA), while xylazine hydrochloride (Rompun) was obtained from Bayer HealthCare AG (Leverkusen, Germany).

## Methods

### Induction, estimation and treatment of glaucoma

This study was approved by the local ethical committee of the Research Institute of Ophthalmology (RIO committee, Giza, Egypt) in accordance with the ARVO statement for the use of animals in ophthalmic and vision research. At the beginning of the experiment, animals were anesthetized by intramuscular injection of a 1:1 mixture of ketamine hydrochloride:xylazine. In the same context, one drop of local numb eye drop (Benox, Eipico Co., Egypt) was applied. The IOP baseline was measured with Schiötz tonometer using 5.5 g and 7.5 g weights. During the experimental period, the daily care regime of rabbits was performed by veterinary consultant and includes the observation of food consumption and fecal characteristics.

**Fig 1:**
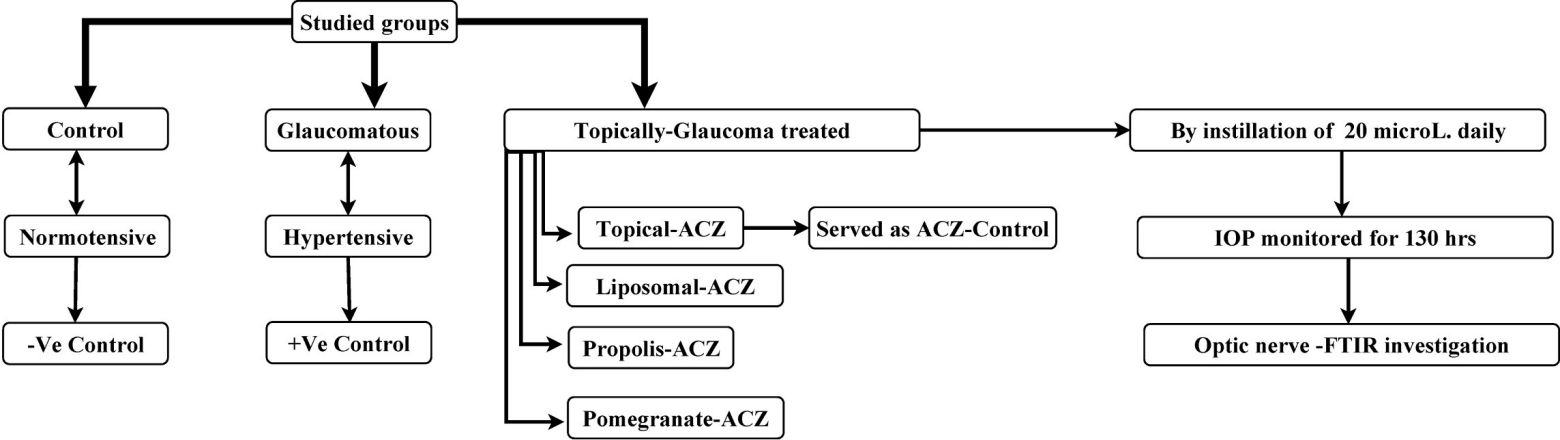
Study design.

Animals were then categorized into sex groups with ten animals each. The first group was served as the normotensive control while the rest of groups were subjected to induced ocular hypertension by subconjunctival injection of glaucocorticoid betamethasone as previously described [18].The induction was achieved by injecting 20 µl once a week and repeated for three weeks. The injected betamethasone suspension contains 2 mg/ml betamethasone sodium phosphate and 5 mg/ml betamethasone dipropionate. The increase in IOP was also monitored by Schiötz tonometer as described before. Four of the hypertensive groups were treated daily by instillation of 20 µl of: topical-ACZ, DPPC-ACZ, propolis-ACZ and pomegranate-ACZ nanopreparations till the end of the study. The last hypertensive group, glaucomatous one, received no treatment and served as a positive control. Study design is provided in Fig 1.

### Propolis and pomegranate extracts

Propolis or pomegranate peel (100 g powder) was dispersed individually in 50 ml of hexane and kept at 4°C for 24 hrs. The suspensions were filtered using Whatman No.1 filter paper followed by successive filtration through 0.8, 0.45 and 0.22 nm syringe filters to remove any debris then, the resultant solutions were dried in a rotary evaporator till complete dryness. The extract was weighted and taken in a known volume of hexane and stored at 4°C for further use. Structural characteristics of extracts were studied by FTIR spectroscopy as well. An aliquot of each extract containing 15 mg of the lipophilic materials was layered on KBr disks and allowed to dry under reduced pressure. The spectra were recorded in the frequency range 4000–400 cm^-1^ using Nicolet is5 instrument (Thermofisher Scientific, MA, USA).

### Preparation of polyphenols rich-Acetozolamide nanoparticles

Ten mg of hexane extracts of either propolis or pomegranate were transferred to a round bottom flask where 10 mg of acetozolamide (ACZ) in acetone:methanol (4:1 v/v) were added. The mixed solution was sonicated for 0.5 min using ultrasonic probe. Removal of the solvents was accomplished via rotary evaporator under reduced pressure and heat. The resulted dry film was kept under reduced pressure overnight to remove any traces of the included solvents. The dried film was then hydrated with 10 ml of ultrapure water and subjected to ultrasonication using ultrasonic probe for 15 min/3 min interval in an ice bath to avoid the heat effect during sonication.

### Preparation of DPPC-ACZ liposomes

Liposomal-ACZ was prepared by dissolving 10 mg of DPPC in ethanol and ACZ (10 mg) was dissolved in acetone:methanol (4:1 v/v). The two preparations were then transferred to a round bottom flask and mixed with ultrasonic probe for 0.5 min. Solvents were then evaporated under reduced pressure and heat using rotary evaporator and kept under reduced pressure overnight to remove any solvent residues if any. The resultant thin film was then hydrated with 10 ml of ultrapure water and vortexed until the lipid film is fully suspended. The suspension was then subjected to 5 cycles of ultrasonication where each cycle lasts for 3 min. Attention was given to heat produced during ultrasonication in order to avoid losing any of the ingredients. The suspension was centrifuged in a cooling bench centrifuge (13,000 rpm) for 30 minutes. Liposomal-ACZ pellet was washed three times, centrifuged and finally resuspended in ultrapure water so that the final concentration of DPPC:water is 1:1 w/v.

### Encapsulation efficiency

One ml of the DPPC-ACZ liposomes was transferred to centrifuge test tube to sediment the liposomes and removal of the suspended liquid. After centrifugation (13,000 rpm for 30 min), liposomes were freeze dried and mixed with one ml of acetone:ethanol (4:1 v/v) in order to perturb the structure and release the encapsulated ACZ. The solution was then centrifuged for 30 min at 25,000 rpm and the supernatant (500 µl) was used to determine the amount of encapsulated ACZ by measuring the absorbance of the solution at 324 nm using thermo-fisher spectrophotometer (evolution 600, USA). Encapsulation efficiency was determined according to a previously established standard curve of ACZ using the same conditions.

### Size distribution and zeta potential measurements

In order to determine the size of the prepared nanoemulations; 50 µl of each preparation separately were diluted to 5 ml of ultrapure water. The size distribution was measured using Zeta PALS system (Brookhaven Instruments Corporation, Holtville, NY, USA)at 25°C. Zeta potential (surface charge) was measured using the same instrument and was monitored for three months.

### Optic nerve and Fourier transform infrared spectroscopy

Animals were sedated as previously mentioned then, euthanized by intraperitoneal injection of sodium pentobarbital (120 mg/kg body weight). Immediately, the optic nerve was dissected and immersed in liquid nitrogen then crushed to powder. This powder (20 mg) was mixed with 80 mg potassium bromide (KBr, IR grade) and pressed by the provided kit from the manufacturing company. The resulted disk was used to record the infrared absorption spectrum using infrared spectophotometer model Nicolet is5 (Thermofisher Scientific, MA, USA). Spectra were recorded at room temperature under continuous flow of nitrogen gas to avoid the effect of atmospheric CO_2_ and water vapor if any. Typically one hundred interferograms were obtained for each sample. The individual spectrum from each animal within the same group was combined to produce a group spectrum that will be analyzed by Fourier deconvolution and displayed in this study.

### Statistical analysis

All results are expressed as the mean±SD. Groups were statistically compared using one way ANOVA and the significance level was set at p<0.05.Spectral analysis was performed using OriginPro software 2015 (Origin Lab Corporation, Northampton, MA, USA).

## Results

### Structural characteristics of extracts

**Fig 2:**
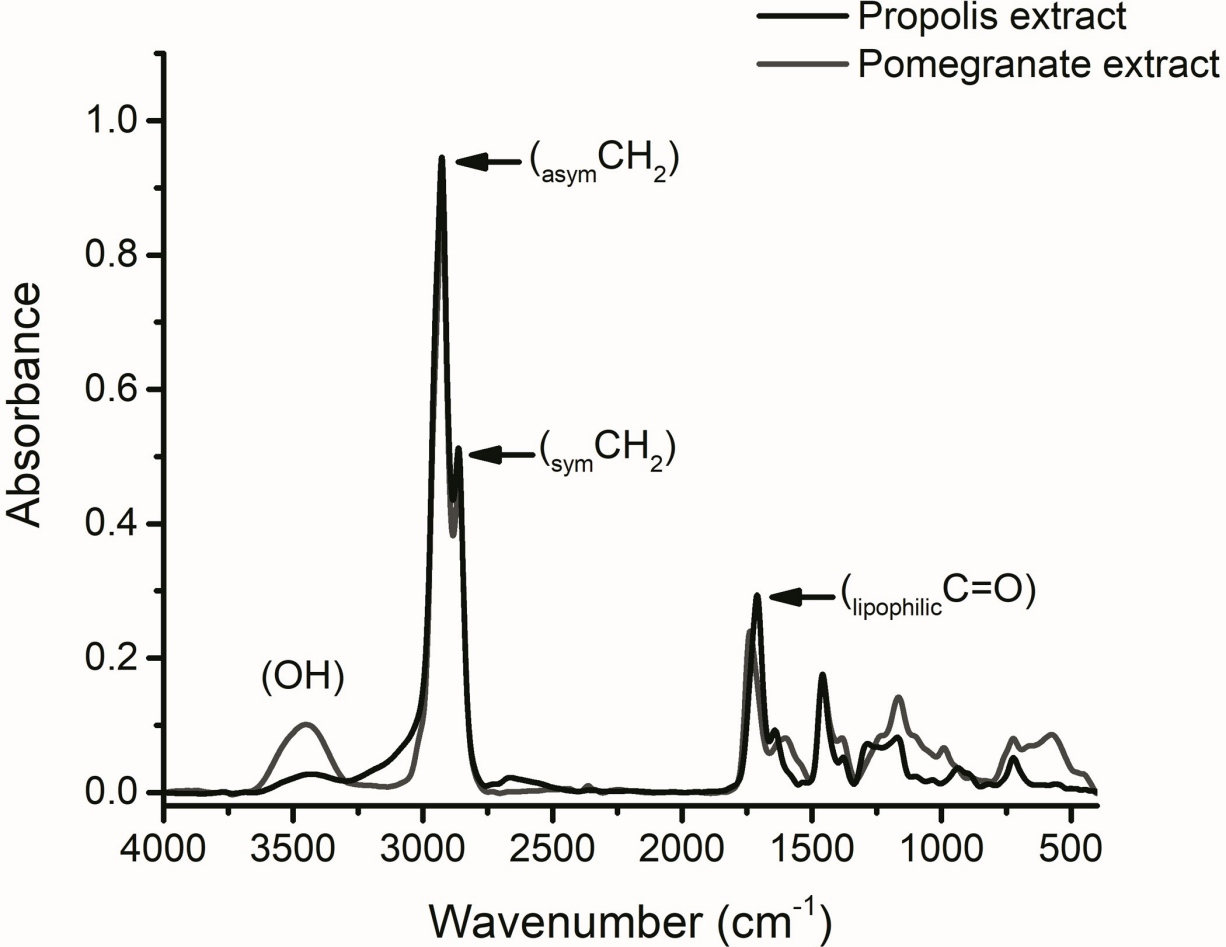
Infrared structural characteristics of propolis and pomegranate peel lipophilic extracts.

Fig 2 displays the infrared absorption pattern of both propolis and pomegranate peel lipophilic extracts. The main features of FTIR-patterns indicate that propolis extract is characterized by higher absorption intensities of hydrocarbon chains as indicated by _asym_CH_2_ (2925±2 cm^-1^), _sym_CH_2_ (2860±3 cm^-1^) and hydrocarbon bonded C = O (1710±1 cm^-1^). Reduced intensity of the hydroxyl bond (OH) absorption is obvious as compared with pomegranate peel absorption pattern.

### Nanopreparation properties

The measured particle size of lipophilic-ACZ preparations was characterized by reduced particle size when compared with liposomes-ACZ; it was 176±10 nm and 190±8 nm corresponding to propolis and pomegranate preparations respectively while, it was found to be 223±6 nm for liposomal-ACZ preparation. On the other hand, all ACZ nanopreparations show negative zeta potential. The average surface charge value over a three months follow up period is -25.8±8, -39.6±7 and -41.3±8 mV for liposomes-ACZ, pomegranate-ACZ and propolis-ACZ respectively. Regarding the liposomal form of ACZ, the calculated encapsulation efficiency is 76%.

### Intraocular pressure (IOP)

**Fig 3:**
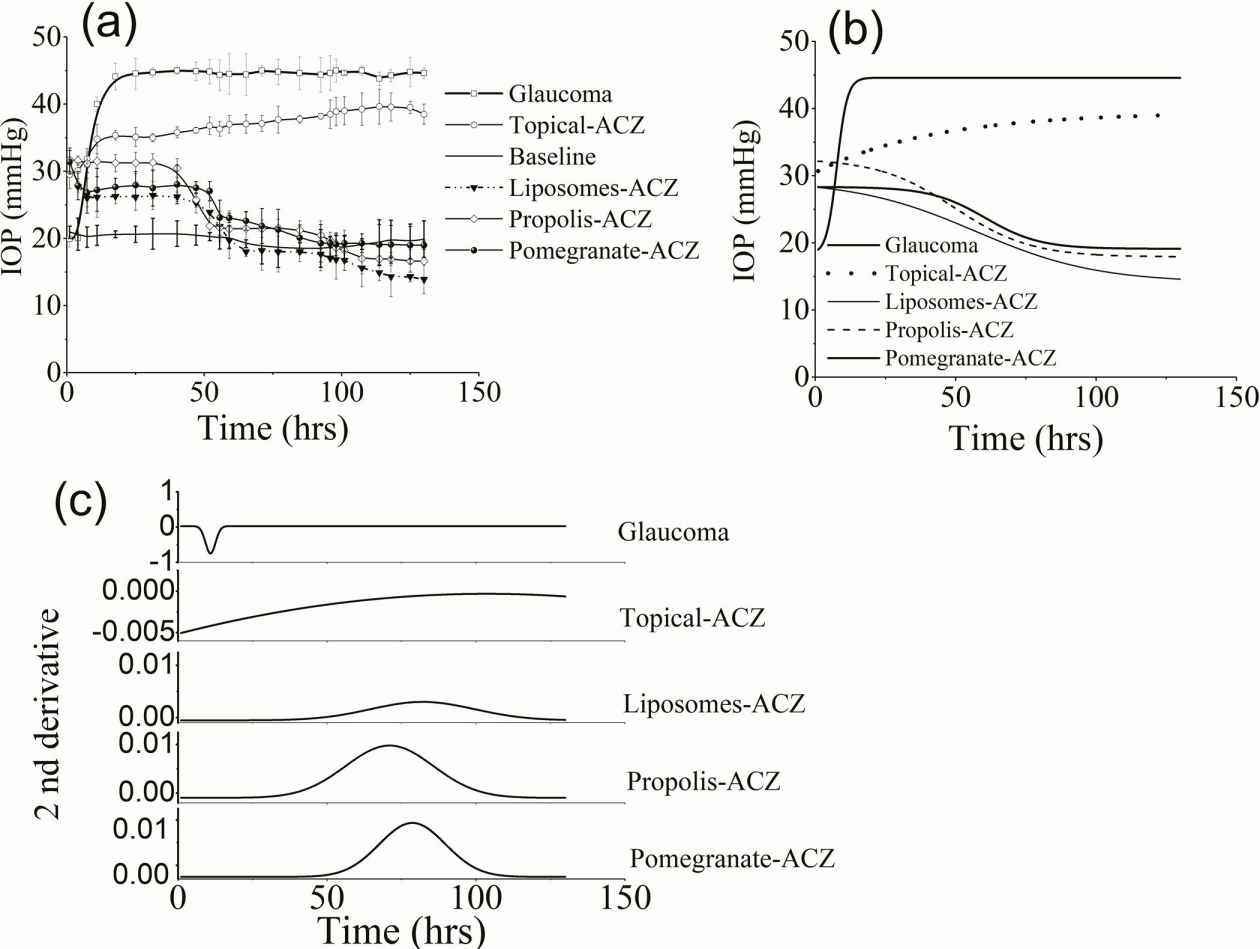
(a) IOP variation in all groups, (b) Dose-response curve fitting and (c) differentiated curves for estimation of pharmacological parameters.

Fig 3A illustrates the variation of the measured IOP over a follow up period of 130 hrs for all groups. The average value of the IOP for the normotensive control is 18.77±3.7 mmHg and for glaucomatous group it was increase to 43.7±3.2 mmHg. The liposomal form of ACZ was found to reduce the IOP to an average value of 14.8±1.3 mmHg (p<0.05). Lipophilic-ACZ nanopreparations were also characterized by significant reduction in the measured IOP where its value was 17.9±1.1 and 19.2±2.6 mmHg for propolis-ACZ and pomegranate-ACZ respectively. Topically applied ACZ was associated by a high IOP (30.8±2.1 mmHg) relative to the normal value.

The data shown in Fig 3A were fitted using dose response fitting function and the results are given in panel b of Fig 3. To determine the pharmacological parameters as the bioavailability i.e, the area under curve (AUC) and the maximum time required to achieve maximum decrease in the measured IOP (t_max_), figures in panel b were differentiated and displayed in panel c of Fig 3 where the resulted parameters are given in Table 1.

**Table 1:** Maximum IOP achieved and the resulted pharmacological parameters due to dose-response curve fitting.

	AUC	t_max_	_max_IOP
Glaucoma	3.4±0.2	10.9±0.1	43.7±2.1
Topical-ACZ	0.04±0.005	103.6±1.5	30.8±2.1
Liposomes-ACZ	^†^0.153±0.07	82.1±2.2	14.8±1.3
Propolis-ACZ	^†^0.4±0.05	70.9±0.7	17.9±1.1
Pomegranate-ACZ	^†^0.30±0.01	78.6±0.3	19.2±2.6

AUC: area under curve and t_max_: maximum time to achieve maximum IOP decrease except for glaucoma group.

^†^ Significant relative to Topical-ACZ.

Induction of glaucoma was achieved after 10.9±0.1 hrs and lasts to the end of the experiment. As shown in Table 1, the fitted AUC was higher for propolis-ACZ followed by pomegranate-ACZ; liposomal-ACZ and finally by topical-ACZ. Treatment with propolis-ACZ nanopreparation was associated with the optimum t_max_ when compared with the other ACZ preparations.

### Optic nerve analysis by Fourier transform infrared spectroscopy

Structural and conformational changes associated with glaucoma and the effect of different treatments was studied by FTIR spectroscopy in the frequency range 4000–900 cm^-1^. Fourier deconvolution was applied to resolve any overlapping of the absorption bands. The spectra are displayed in two ranges: 4000–2500 cm^-1^ which represents the stretching of NHOH and CH bonds, and 2000–900 cm^-1^ that reflects the fingerprint region.

### NHOH and CH stretching region

**Fig 4:**
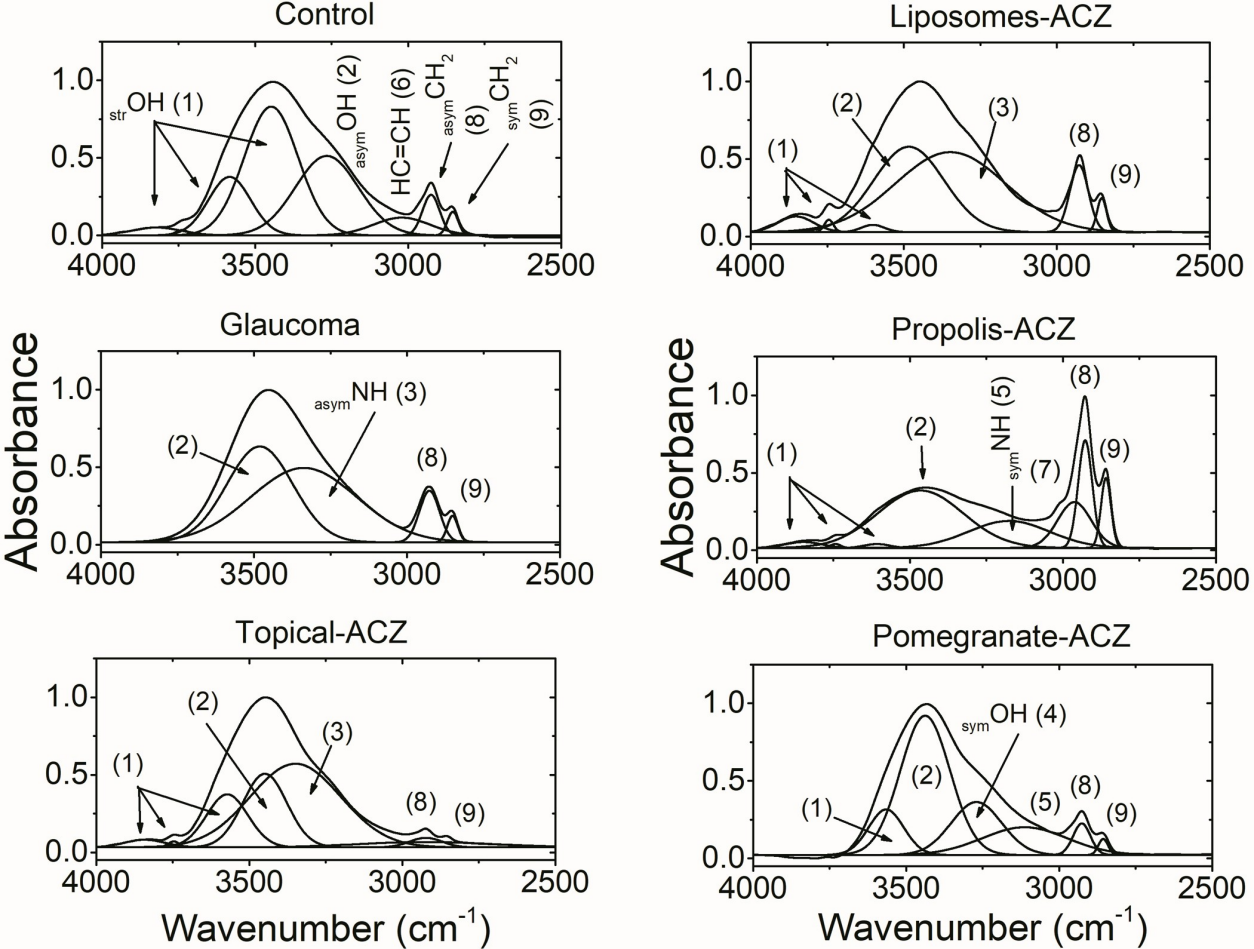
Characteristic absorptions of NHOH-CH bonds and the deconvoluted (underlying) bands. Numbers above the bands is to facilitate their assignments.

Fig 4 and Tables 2 and 3 show the characteristic vibrational modes of the NHOH and CH bonds for the control and the other studied groups. Upon deconvolution, the control pattern was characterized by eight absorption bands. This number was markedly decreased to four in glaucoma group where the _str_OH mode of vibration, _sym_OH and the unsaturated HC = CH were restricted. In addition to this observation, the _asym_OH vibrational mode show significant increase in its band position and bandwidth and, _asym_NH vibrational mode is detected.

**Table 2:** Deconvoluted bands in the NHOH region (4000–3000 cm^-1^) for all studied groups.

	_str_OH				_asym_OH	_asym_NH	_sym_OH	_sym_NH
Control	3823±2 189±5	3738±2 163±8		3584±1 163±7	3447±2 208±10		3266±3 242±10	
Glaucoma					^†^3481±3 ^†^254±8	3348±1 408±11		
Topical-ACZ	^†^3842±2 ^†^155±4	^†^3747±3 ^†^42±6		^†^3572±1 ^†^42±5	3449±3 ^†^169±7	3344±1 347±9		
Liposomes-ACZ	^†^3853±1 ^†^136±4	^†^3746±2 ^†^35±7	3602±1 88±2		^†^3484±1 ^†^271±9	3348±2 425±10		
Propolis-ACZ	^†^3848±3 ^†^140±6	^†^3745±2 ^†^38±4	3608±2 97±7		^†^3465±2 ^†^327±6			3176±1 311±12
Pomegranate-ACZ				^†^3566±2 ^†^135±6	^†^3470±1 ^†^171±8		^†^3270±3 194±9	3113±2 325±10

^†^Statistical significance according to the control. Numbers in the first line of each cell represent the wavenumber (cm^-1^) while, those of the second line indicates the bandwidth (cm^-1^).

**Table 3:** Optic nerve stretching vibrational modes of CH bond.

	HC = CH	_asym_CH_3_	_asym_CH_2_	_sym_CH_2_
Control	3020±2 216±5		2925±3 66±8	2854±3 41±9
Glaucoma			2927±2 77±9	2852±2 40±7
Topical-ACZ			2923±3 ^†^125±8	2842±3 ^†^523±12
Liposomes-ACZ			2928±4 72±10	2853±2 37±6
Propolis-ACZ		2963±2 129±7	2927±4 54±9	2859±3 38±5
Pomegranate-ACZ			2926±2 67±11	2856±3 37±8

^†^Statistical significance according to the control. Numbers in the first line of each cell represent the wavenumber (cm^-1^) while, those of the second line indicates the bandwidth (cm^-1^).

Topically applied ACZ by itself was associated with seven detected bands. The higher frequency bands of the _str_OH mode of vibration discernible at 3842±2 cm^-1^ and 3747±3 cm^-1^show a significant increase in their position and a significant decrease in their bandwidth as compared with the control pattern. The lower frequency band of the _str_OH mode was detected at 3572±1 cm^-1^with decreased position and bandwidth. The bandwidth of _asym_OH is decreased and _asym_NH can be detected as well.

The optic nerve of the animal group that treated with liposomal encapsulated ACZ was characterized by seven stretching vibrational bands. The two- higher frequency- bands of the _str_OH bond show the same observation as those of the topically applied ACZ; increased band position and decreased bandwidth. This was concomitant with the detection of a third higher frequency band at 3602±1 cm^-1^. A significant increase in the band position as well as the bandwidth of the _asym_OH bond was observed as compared with the control. Again, _asym_NH vibrational mode can be observed.

Treating glaucomatous eyes with propolis-ACZ show a different FTIR absorption pattern where eight bands were detected as the control pattern but with different assignment and characteristics. Regarding the _str_OH vibrational mode, three bands are obvious and characterized by higher vibrational frequency and reduced bandwidth relative to the control. As previously noticed in the liposomal-ACZ treated group, the _asym_OH mode of vibration also characterized by a significant increase in its band position as well as bandwidth. The _sym_OH vibrational mode that was detected in the control pattern was restricted in this group and instead, the _sym_NH vibrational mode was detected.

The last nanopreparation; pomegranate-ACZ, under investigation show another and different FTIR absorption pattern where six bands were detected. One band is detected in the _str_OH range and characterized by reduced band position and bandwidth as well. The _asym_OH vibrational mode show higher band position and reduced bandwidth relative to the control pattern. In the same context, the _sym_OH vibrational mode was detected with decreased bandwidth and _sym_NH vibrationas well.

Regarding the CH vibrational characteristics of the optic nerve from all studied groups (Table 3), three observations can be noticed; the first one is related to topically applied ACZ where the bandwidth of both _asym_CH_2_ and _sym_CH_2_ was significantly increased; and the second observation is related to the propolis-ACZ treated group where _asym_CH_3_ vibrational mode can be detected. The last observation which related to the rest of the studied group is the unchanged position and bandwidth of CH_2_ vibrational modes.

### Fingerprint region

**Fig 5:**
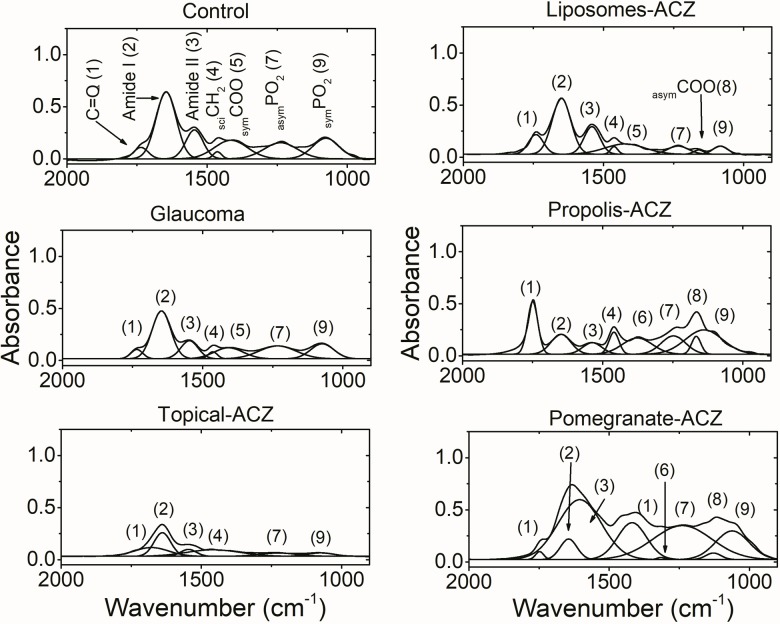
Bending vibrational modes in the frequency range 2000–900 cm^-1^ for all studied groups. Numbers above the bands is to facilitate their assignments.

In Fig 5, the ester carbonyl vibrational mode (C = O) of lipids was detected at 1735±2 cm^-1^ in the control and remains unchanged in the glaucoma group. Treating glaucoma with topical ACZ reduces the frequency of this band to 1678±2 cm^-1^ while, its nanopreparations treatment were associated with significant increase in the band position. On the other hand, its bandwidth was significantly increased due to topical treatment with ACZ and this observation was reversed with lipophilic extracts-ACZ treatment while, remains unchanged in the both glaucomatous group and liposomal-ACZ treated one as given in Table 4.

**Table 4:** Characteristics of different bending vibrations of the fingerprint region for all studied groups.

	C = O	Amide I	Amide II	_sci_CH_2_	_sym_COO	_roc_CH_3_	_asym_PO_2_	_asym_COO	_sym_PO_2_
Control	1735±2 60±5	1646±3 84±8	1545±2 72±10	1463±1 34±7	1412±2 34±10		1238±3 139±10		1076±2 111±9
Glaucoma	1735±1 50±7	1647±2 78±5	1547±2 72±7	1464±2 38±8	^†^1407±3 ^†^121±8		^†^1232±2 154±8		1073±2 97±10
Topical-ACZ	^†^1678±2 ^†^144±4	1641±3 70±6	1546±2 65±9	^†^1457±2 ^†^225±5			^†^1227±2 159±8		1075±3 112±6
Liposomes-ACZ	^†^1741±1 63±4	1650±3 78±7	^†^1539±1 66±2	1462±2 31±5	^†^1423±1 ^†^168±9		^†^1234±2 ^†^87±7	1160±3 42±9	^†^1083±1 ^†^61±5
Propolis-ACZ	^†^1749±3 ^†^44±6	1648±2 83±4	^†^1537±2 80±7	1460±3 42±6		1376±1 125±4	^†^1248±2 ^†^103±5	1166±1 39±12	^†^1138±2 ^†^156±8
Pomegranate-ACZ	^†^1748±2 ^†^34±4	1645±1 73±7	^†^1605±3 ^†^181±8		^†^1417±1 ^†^122±8	1315±2 31±7	1239±3 ^†^246±9	1129±2 64±10	^†^1062±2 ^†^140±4

^†^Statistical significance according to the control. Numbers in the first line of each cell represent the wavenumber (cm^-1^) while, those of the second line indicates the bendwidth (cm^-1^).

The position of protein amide I band that detected at a vibrational frequency of 1646±3 cm^-1^ in the control pattern was not affected by the induced glaucoma or any mode of treatment, in addition its bandwidth nonsignificantly changed between groups. A detailed analysis of this band is given at the end of this section.

Amide II band of protein (1545±2 cm^-1^ in the control pattern) was affected by the treatment with different ACZ nanopreparations; the position of the band was reduced after treating glaucoma with liposomal-ACZ and its propolis preparation while, it increased as a result of treatment with pomegranate-ACZ preparation, this preparation also affecting the vibrational motion around amide II band as indicated in Table 4 by the increased bandwidth.

The bending vibrational mode (_scissoring_CH_2_)that was detected at a vibrational frequency of 1463±1cm^-1^ in the control optic nerve and has a bandwidth of 34±7 cm^-1^ does not affected by the induced glaucoma or after treating it with either liposomes-ACZ or propolis-ACZ preparations. Treatment with topical-ACZ was associated with reduced band position and broad bandwidth; in the same context this mode of vibration was restricted due to treatment with pomegranate-ACZ nanopreparation.

Glaucoma was found to reduce the vibrational frequency of the _sym_COO vibrational mode as compared with the control. Treating glaucoma with topical-ACZ or propolis nanopreparation restrict this vibration while, liposomes-ACZ and pomegranate-ACZtreatments increase both the band position and bandwidth relative to the control.

Two new vibrational modes were detected in the fingerprint region namely _rock_CH_3_ and _asym_COOC. These two vibrational modes were detected after treating glaucoma with lipophilic nanopreparations of ACZ. Liposomes-ACZ treatment was associated with _asym_COOC vibrational mode only.

The phosphate vibrational modes were detected in the control optic nerve at 1238±1 cm^-1^ (_asym_PO_2_) and 1076±2 cm^-1^ (_sym_PO_2_). The former mode is sensitive to all treatments; its band position was reduced after induction of glaucoma and after treating it with topical-ACZ and liposomal-ACZ while, increased due to treatment with propolis-ACZ nanopreparation. The bandwidth of this vibrational mode was also fluctuated after treatment with different nanopreparations of ACZ. The symmetric vibration mode of PO_2_ band was affected after treating glaucoma with nanopreparations only and includes both band position and bandwidth as well.

**Fig 6:**
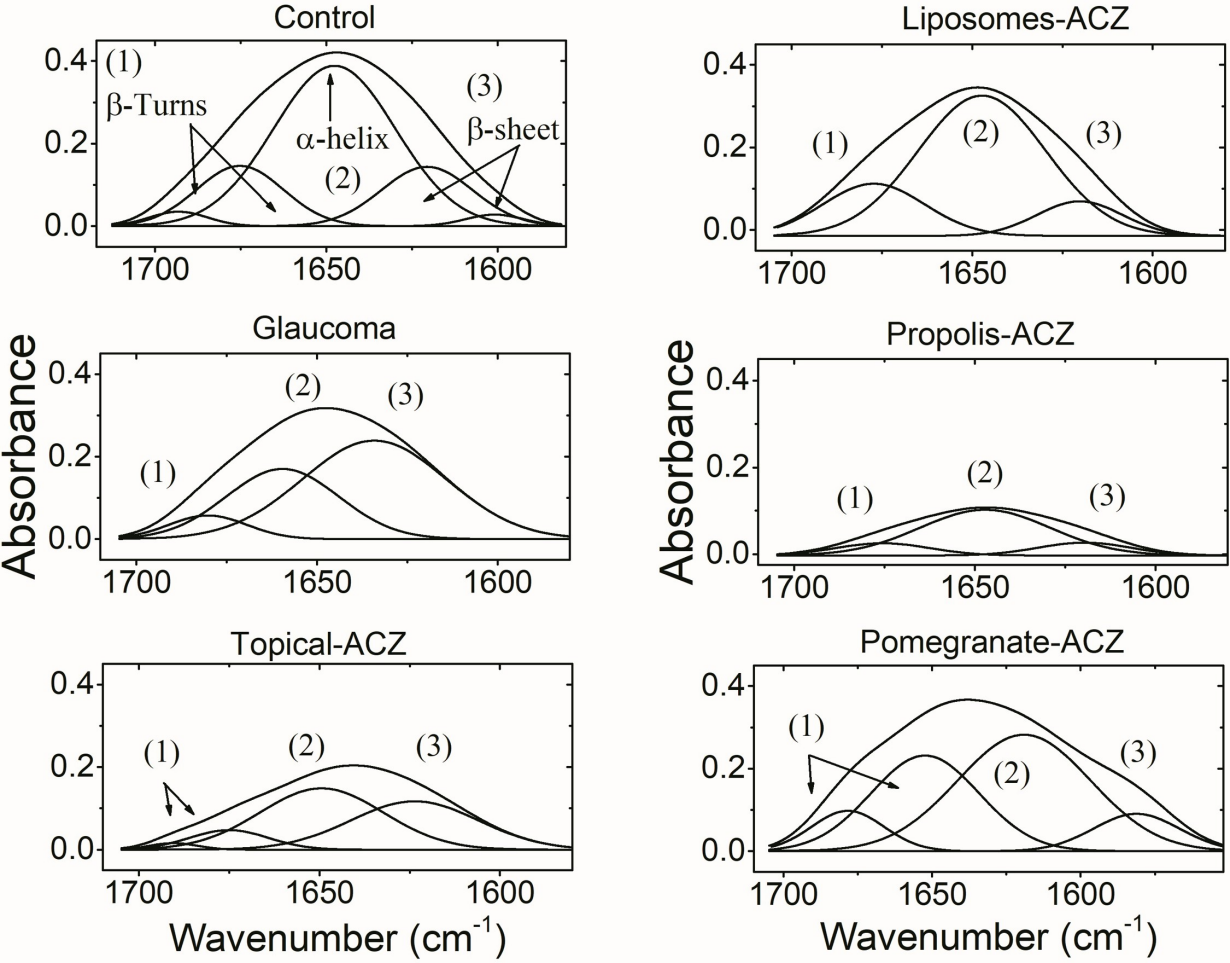
Analysis of amide I band by Fourier deconvolution showing protein secondary structure components. Numbers above the bands is to facilitate their assignments.

The extensive study of amide I band using the curve enhancement procedure (Fig 6) is used to study the protein secondary structure. Five protein structural components were resolved in the control band and correspond to β-turns, α-helix and β-sheet. The induced glaucoma was associated by three structural components while, topically applied ACZ and pomegranate-ACZ treatment were characterized by four protein secondary structural components. Treating glaucoma with either liposomal form of ACZ or propolis-ACZ nanopreparations was associated with three structural components. The area percentage of these different structural components is used to indicate the concentration of that component. Accordingly and as given in Table 5, glaucoma reduces the α-helix content of the optic nerve and β-turns while increases the content of β-sheet as compared with the control. Treatment with topical ACZ was characterized by the same observations as the glaucoma group except for turns. Liposomes-ACZ and propolis-ACZ preparations show protein secondary structures contents that mimics the control values. When pomegranate-ACZ preparation was topically applied, it was found that the protein secondary structural components resemble those of glaucoma group.

**Table 5:** Estimated protein secondary structural components of the optic nerve dissected from all groups expressed as area percentage.

	α-helix	β-sheets	Turns
Control	61.9±9	19.2±6	19.1±5
Glaucoma	^†^32.9±6	^†^59.5±8	^†^7.6±4
Topical-ACZ	^†^46.6±4	^†^39.6±7	13.8±3
Liposmes-ACZ	68.8±6	12.4±7	12.8±2
Propolis-ACZ	71.9±8	14.4±6	13.7±4
Pomegranate-ACZ	^†^32.4±5	^†^58.6±5	^†^9±3

^†^ Significance relative to the control group

## Discussion

Glaucoma is a multifactorial disease and its pathogenesis can be related to either a mechanical damage due to increased IOP or vascular dysregulation that affects the trabecular meshwork [19]. Therefore, decreasingIOP is essential to prevent the development or progression of glaucoma. The results of this study (Fig 3) confirm the limited efficiency of topically applied ACZ in lowering the IOP that previously reported in the literature due to two major obstacles; poor aqueous solubility and low permeability coefficient[3–4]while, DPPC-encapsulated ACZ and the lipophilic extract-nanopreparations greatly improve its hypotensive efficiency.

One method of making comparisons among different doses of the same drug is to compute the area under the curve (AUC) which reflects the concentration of that drug and is used as a measure of drug exposure i.e. its bioavailability. Accordingly, propolis-ACZ is associated by the highest bioavailability among different ACZ preparations included in this study and it was concomitant with the best t_max_ obtained. The contradicting results of acetozolamide and its polyphenols-rich nanopreparations in decreasing the increased IOP are providing evidence that propolis and pomegranate extracts have antiglaucoma properties. Acetozolamide lowering effect is attributed to its ability of inhibiting the aqueous humor production. Therefore, plant extracts could exerts their hypotensive effects via reducing the production of aqueous humor and/or protecting the trabecular meshwork from oxidative stress associated with damaging the structural integrity of trabecular meshwork, thus preserving the eye's endogenous antioxidant system. On the other hand, liposomal-ACZ results show that liposomes are able to smuggling ACZ behind the normal ocular barriers, thus exerting its hypotensive effect. Note that, topical instillation of drugs into the eye is a convenient-preferred and non-invasive method of administration due to its direct and localized effects as well as the availability of the therapeutic drug in high concentration. These advantages ensure that the incidence of side effects due to systemic administration of ACZ is minimized.

Non-essential phytoconstituents associated with pomegranate peel or propolis powder are different and in turn may display different functions. Pomegranate peel, in general, is a rich source of more than 300 phytochemicals. Egyptian pomegranate peel contains a total phenolic content of 17.24±1.11 mg gallic acid equivalent/g sample and a total flavoniods of 34.28±1.47 mg rutin equivalent/g sample where, the dominant phenolic constituent is gallic acid (5.43 mg/g) [20]. Oleic acid and Coumaric acid are the most abundant aliphatic and aromatic acids respectively. Propolis, on the other hand, contains more than 300 bioactive components [21] as pomegranate peel but, Egyptian propolis showed reduced concentrations of the previously mentioned phytoconstituents of pomegranate peel. The amount of total phenolic content was found to be 0.138±0.003 mg gallic acid equivalent/g sample and that of total flavonoids was 0.75 mg /g sample. Hexadecyl caffeate is the abundant form of esters while cycloartinol and β-amyrine are the abundant triterpens[21–22].

Iomdina et al. 2015 [23]show that elevated IOP may have a direct impact on mitochondria; the primary source of reactive oxygen species. Therefore, increased mitochondrial oxidative stress contributed to apoptosis and development of glaucoma. The antioxidant activity of propolis phenolics is mainly due to their redox properties, which allow them to act as reducing agents, hydrogen donators, and singlet oxygen quenchers. In addition, they have a metal chelation potential. However, the decrease of the phenolic compounds content is not accompanied by a decrease of its activity. The triterpenes, consisting about 18% of Egyptian propolis and might play a role in its antioxidant activity [24]. Propolis-ACZ is more effective neuroprotector as it maintains the normal secondary structure of optic nerve protein and this can be directly linked to its higher content of hydrocarbon chains (Fig 2). Protein insolubility is dependent on the content of the β-sheet structure. The association between the decreased α-helix content and increased β-sheet content is indicative of expression of a new protein structure with different compositional characteristics or structural rearrangement of the already existing proteins. This gives the impetus that protein tends to form aggregates as it can be noticed in the glaucomatous group, topical-ACZ treated group and pomegranate-ACZ treated one as well. The interesting finding that liposomal-ACZ exerts protective effect on the optic nerve protein secondary structure supports the mechanical theory of glaucoma side effects.

Regarding the structural and conformational characteristics of the optic nerve; the position of the ester bond (C = O) of lipids was used to probe the length of the fatty acid chainwhere longer fatty acid chain length results in shift to higher wavenumbers[25]. Hence, topical-ACZ treatment was noticed to be associated with shorter fatty acid chains and, when it encapsulated in liposomes or associated with lipophilic extracts, it was associated with longer fatty acid chains. On the other hand, the degree of freedom (motion) around this bond that detected by its bandwidth reflects the vibrational motion around the interface region of the phospholipids[26]. Our results showed that this motion is greatly affected by the way ACZ is introduced. It was found that lipophilic extract-ACZ nanopreparations increase the vibrational motion around that band (Fig 5 and Table 4), and this is contradicting the effect of topical-ACZ. Liposomal-ACZ does not affect the vibrational motion around the phospholipid interface region.

When molecules rotate around single bond they called rotameres and are indicative for the conformation changes within the lipid hydrocarbon chains. The relative amount of rotameres was calculated according to the vibrational frequency of_asym_CH_2_ as previously described[27]. According to the results given in Table 3, the % gauche rotamers of hypertensive groups whether treated with hypotensive nanopreparations or not was found to be 1.1±0.5 and that of normotensive control is 0.8±0.2. This is an indication that no change in the conformation of the lipid hydrocarbons. But, the careful analysis of the vibrational characteristics of the hydrocarbon chains indicates other behavior. The area percentage ratio for _asym_CH_2_/_sym_CH_2_ reflects the degree of disorder within the lipid hydrocarbon chain[28–30]. This ratio was increased from 1.42±0.6 in the normotensive control to 3.7±0.4, 3.93±0.8 and 3.5±0.9 in glaucomatous, liposomal-ACZ and pomegranate-ACZ respectively while, dramatically decreased (0.432±0.1) in the topically applied ACZ. Propolis-ACZ nanopreparation does not affect the order state of lipid hydrocarbon chains (2.2±0.8).In addition, the bending vibrational mode of CH_2_ bond in the fingerprint region is used to monitor the acyl chain packing of the hydrocarbon chains [30]. This acyl chain packing is affected by the treatment with topical-ACZ and pomegranate-ACZ only.

## Conclusion

Glaucoma does not affect the interface region of the phospholipids, while ACZ affecting it and this is depends on the way ACZ is introduced. The fatty acid structural characteristics of the optic nerve phospholipids were characterized by longer length after treatment with lipophilic-ACZ and liposomes-ACZ nanopreparations and this is in contrast to the topical-ACZ. Altogether led to the conclusion that the studied nanopreparations have hypotensive effects and, propolis-ACZ is a promising neuroprotective formulation.

## Supporting information

S1 File NC3RS ARRIVE guidlines checklist. (PDF)

S2 File Size distribution data for liposomal-ACZ nanopreparations. (TXT)

S3 File Size distribution data for pomegranate-ACZ nanopreparations. (TXT)

S4 File Size distribution data for propolis-ACZ nanopreparations. (TXT)

S1 Fig Study design. (TIF)

S2 Fig Infrared structural characteristics of propolis and pomegranate peel lipophilic extracts. (TIF)

S3 Fig (a) IOP variation in all groups, (b) Dose-response curve fitting and (c) differentiated curves for estimation of pharmacological parameters.
(TIF)

S4 Fig Characteristic absorptions of NHOH-CH bonds and the deconvoluted (underlying) bands. Numbers above the bands is to facilitate their assignments.
(TIF)

S5 Fig Bending vibrational modes in the frequency range 2000–900 cm^-1^ for all studied groups. Numbers above the bands is to facilitate their assignments.
(TIF)

S6 Fig Analysis of amide I band by Fourier deconvolution showing protein secondary structure components. Numbers above the bands is to facilitate their assignments.
(TIF)
