# Supplementary figures and images for: Oculohypotensive effects of various acetozolamide nanopreparations for topical treatment of animal model-induced glaucoma and their impact on optic nerve
Source: PLoS One. 2019 Feb 21;14(2):e0212588. doi: 10.1371/journal.pone.0212588 (PMC6383913; doi:10.1371/journal.pone.0212588)

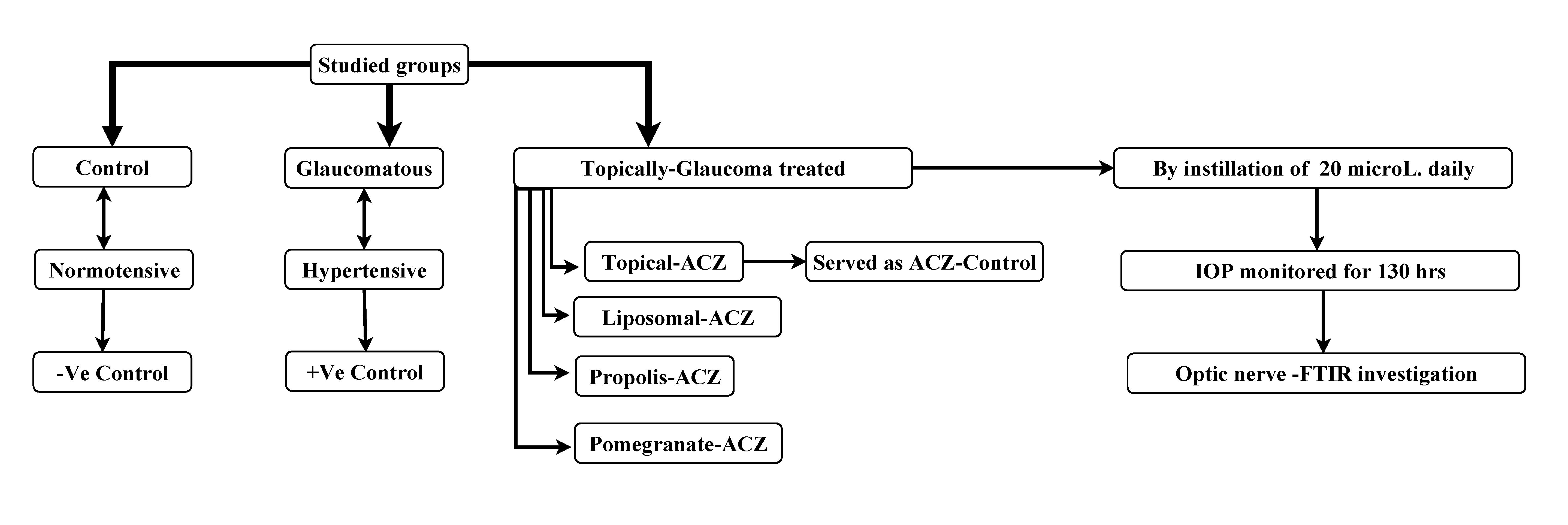

Supplement: S1 Fig — (TIF) [file pone.0212588.s005.tif]

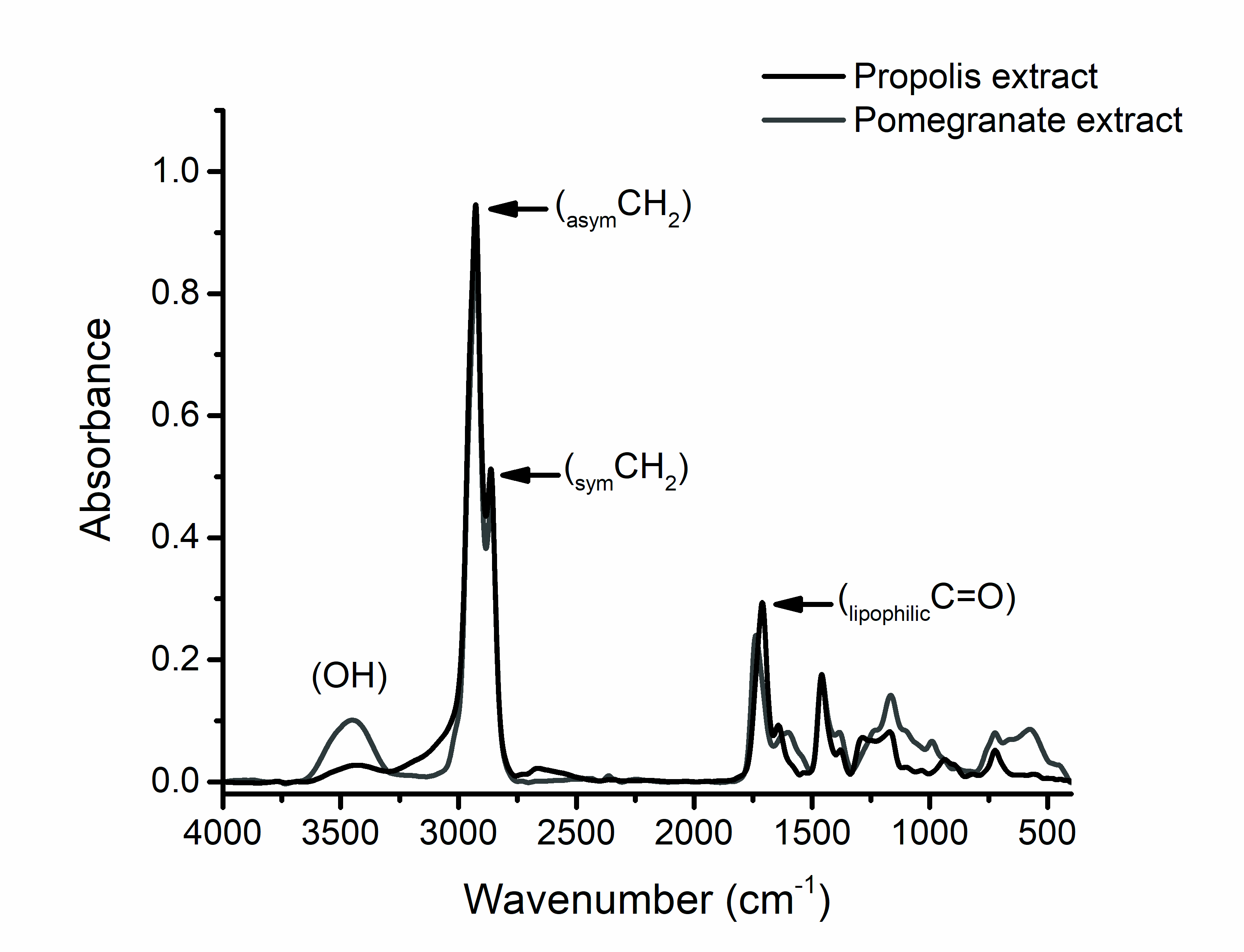

Supplement: S2 Fig — (TIF) [file pone.0212588.s006.tif]

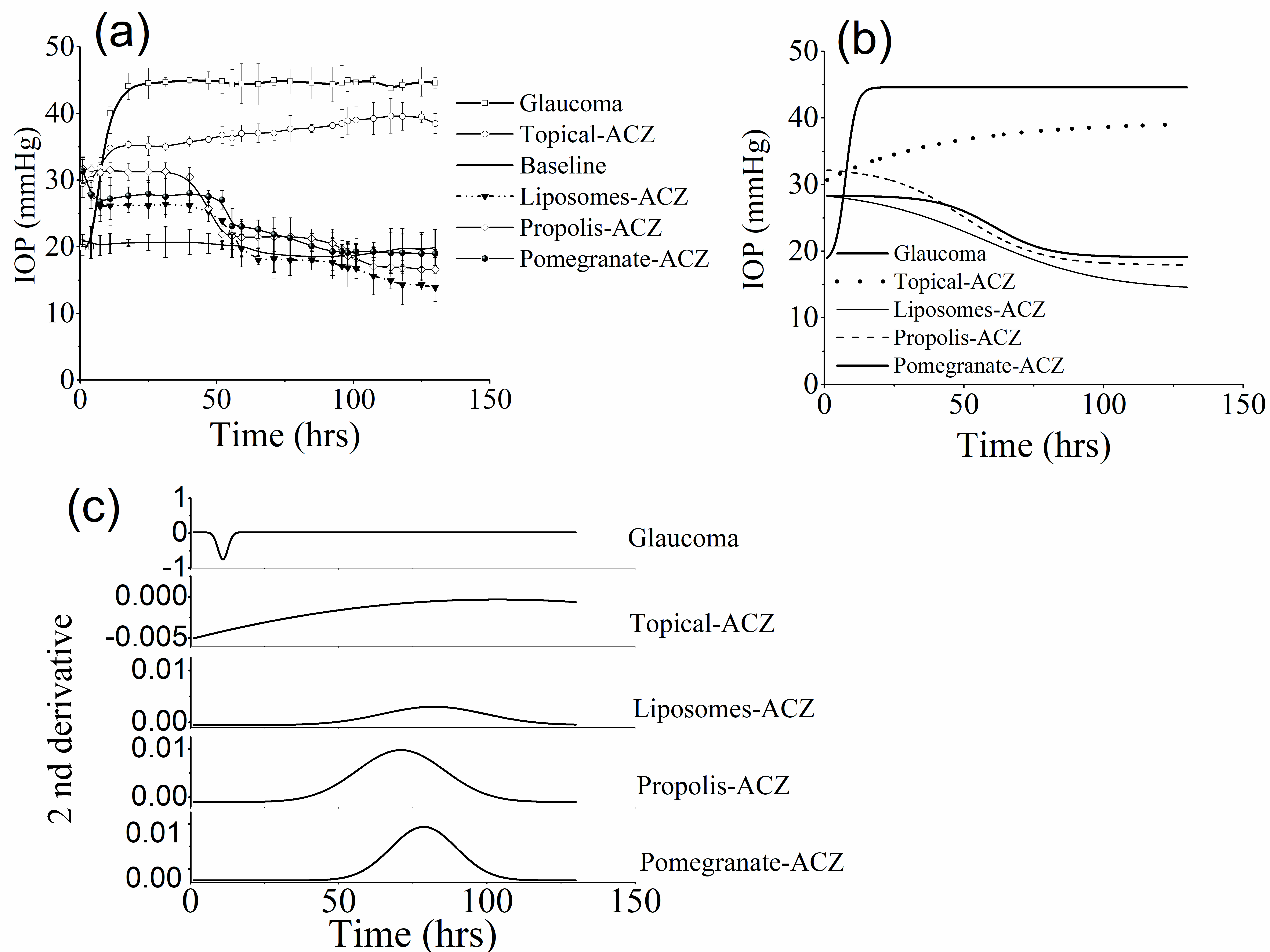

Supplement: S3 Fig — (a) IOP variation in all groups, (b) Dose-response curve fitting and (c) differentiated curves for estimation of pharmacological parameters. (TIF) [file pone.0212588.s007.tif]

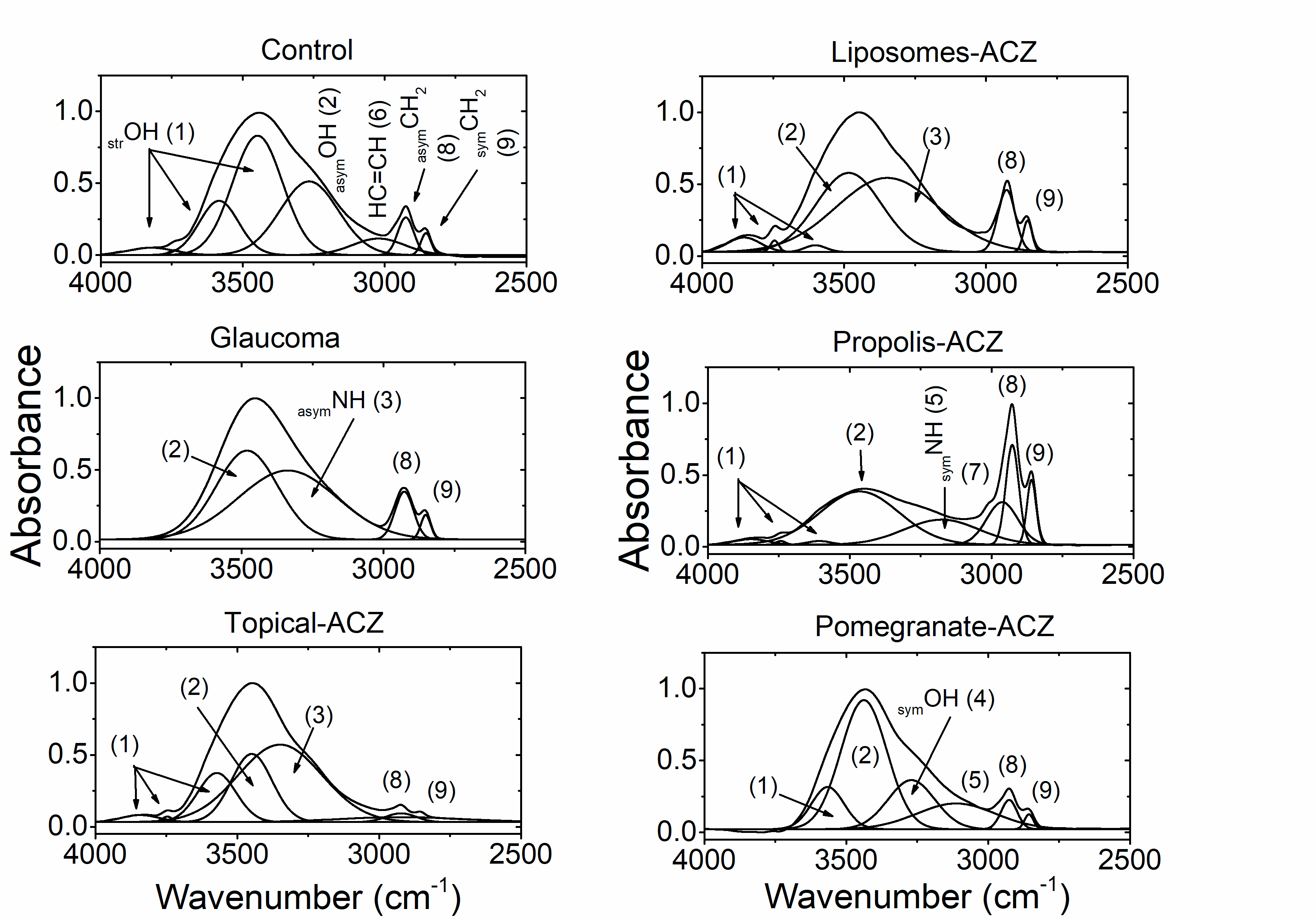

Supplement: S4 Fig — Numbers above the bands is to facilitate their assignments. (TIF) [file pone.0212588.s008.tif]

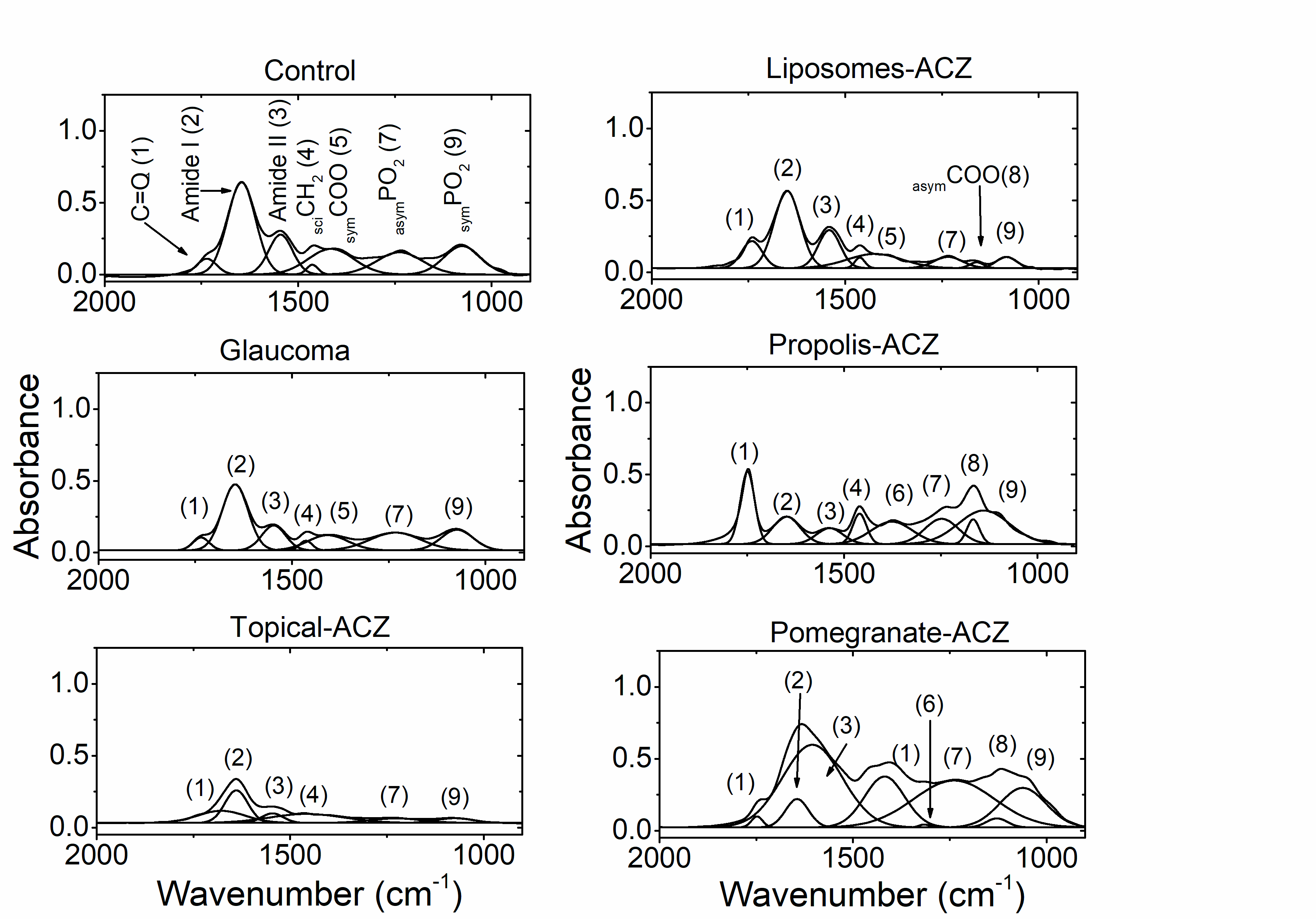

Supplement: S5 Fig — Numbers above the bands is to facilitate their assignments. (TIF) [file pone.0212588.s009.tif]

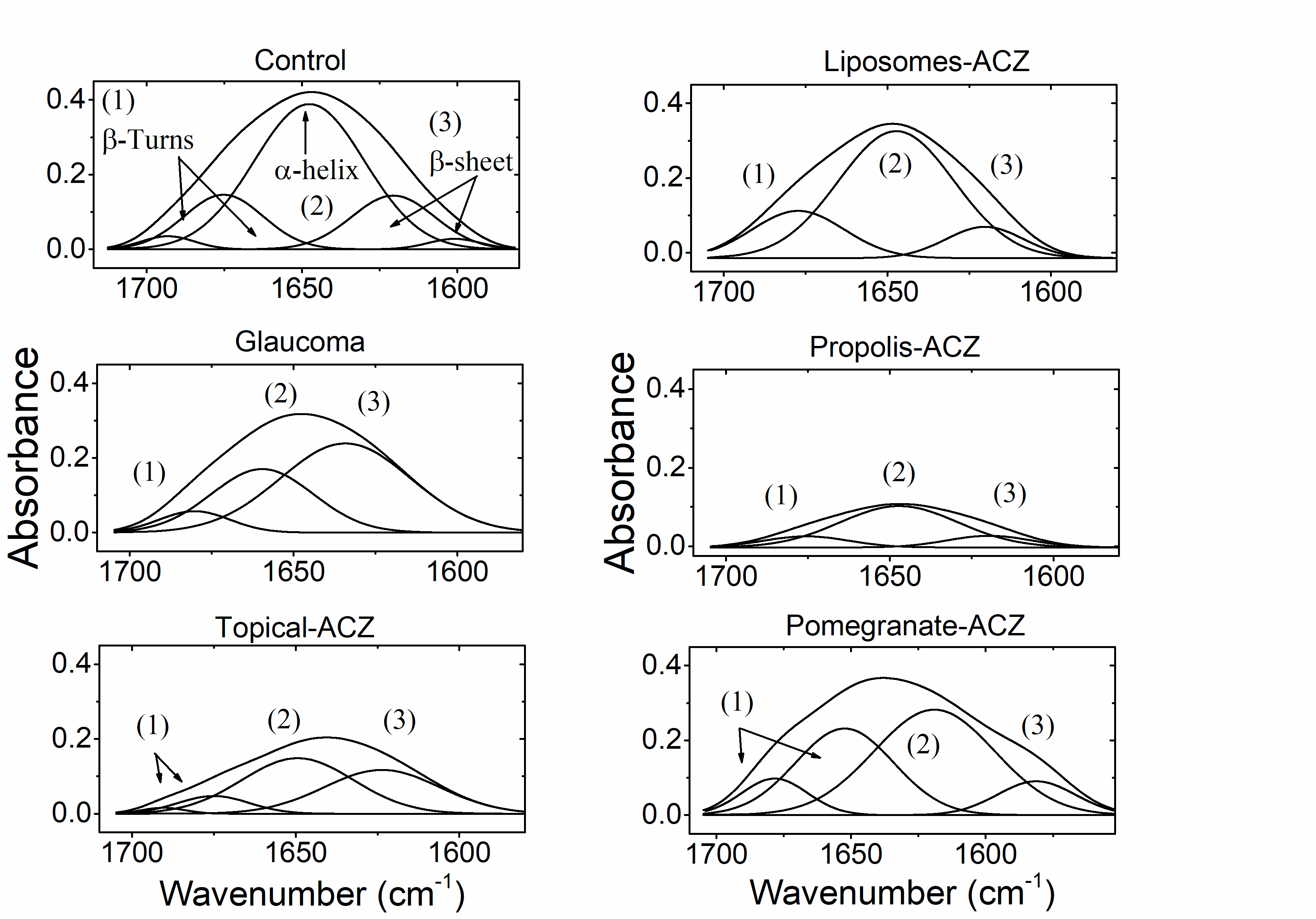

Supplement: S6 Fig — Numbers above the bands is to facilitate their assignments. (TIF) [file pone.0212588.s010.tif]
